# Engineered Lysins With Customized Lytic Activities Against Enterococci and Staphylococci

**DOI:** 10.3389/fmicb.2020.574739

**Published:** 2020-11-25

**Authors:** Hana Sakina Binte Muhammad Jai, Linh Chi Dam, Lowella Servito Tay, Jodi Jia Wei Koh, Hooi Linn Loo, Kimberly A. Kline, Boon Chong Goh

**Affiliations:** ^1^Antimicrobial Resistance Interdisciplinary Research Group, Singapore-MIT Alliance for Research and Technology Centre, Singapore, Singapore; ^2^Singapore Centre for Environmental Life Sciences Engineering, School of Biological Sciences, Nanyang Technological University, Singapore, Singapore

**Keywords:** lysin, *Enterococcus*, *Staphylococcus*, endolysin, antibiotic alternatives, antimicrobial resistance, domain swapping

## Abstract

The emergence of multidrug-resistant bacteria has made minor bacterial infections incurable with many existing antibiotics. Lysins are phage-encoded peptidoglycan hydrolases that have demonstrated therapeutic potential as a novel class of antimicrobials. The modular architecture of lysins enables the functional domains – catalytic domain (CD) and cell wall binding domain (CBD) – to be shuffled to create novel lysins. The CD is classically thought to be only involved in peptidoglycan hydrolysis whereas the CBD dictates the lytic spectrum of a lysin. While there are many studies that extended the lytic spectrum of a lysin by domain swapping, few have managed to introduce species specificity in a chimeric lysin. In this work, we constructed two chimeric lysins by swapping the CBDs of two parent lysins with different lytic spectra against enterococci and staphylococci. We showed that these chimeric lysins exhibited customized lytic spectra distinct from the parent lysins. Notably, the chimeric lysin P10N-V12C, which comprises a narrow-spectrum CD fused with a broad-spectrum CBD, displayed species specificity not lysing *Enterococcus faecium* while targeting *Enterococcus faecalis* and staphylococci. Such species specificity can be attributed to the narrow-spectrum CD of the chimeric lysin. Using flow cytometry and confocal microscopy, we found that the *E. faecium* cells that were treated with P10N-V12C are less viable with compromised membranes yet remained morphologically intact. Our results suggest that while the CBD is a major determinant of the lytic spectrum of a lysin, the CD is also responsible in the composition of the final lytic spectrum, especially when it pertains to species-specificity.

## Introduction

Antimicrobial resistance (AMR) is on the rise worldwide, which poses a public health crisis given the slow pace of antibiotic development. To treat infections caused by drug-resistant bacteria, health care providers are resorting to mothballed antibiotics that come with serious side effects (Ventola, 2015; Velez and Sloand, 2016). Therefore, there is a dire need to develop novel antibacterial agents. Lysins derived from bacteriophage present a promising alternative to antibiotics as they are less prone to resistance and can kill bacteria selectively and rapidly (Fischetti, 2008, 2018; Zou et al., 2011; Schmelcher et al., 2012; Maciejewska et al., 2018; Oliveira et al., 2018).

Lysins are phage-encoded peptidoglycan hydrolases that cleave the bonds in peptidoglycan chains of the bacteria cell wall. When applied exogenously to bacteria as purified recombinant proteins, lysins rapidly hydrolyze the peptidoglycan layer, resulting in cell lysis and bacterial death. Hence, lysins are particularly effective against Gram-positive bacteria as the peptidoglycan layer is accessible extracellularly due to the absence of an outer membrane, making the peptidoglycan layer accessible from the outside (Hermoso et al., 2007; Fischetti, 2008, 2010). Lysins targeting Gram-positive bacteria contains two functional, modular domains, namely catalytic domain (CD) and cell wall binding domain (CBD). CD cleaves the specific bonds of the bacterial peptidoglycan, and CBD recognizes the target bacteria and anchors the lysin by binding to specific carbohydrate ligands on the bacterial cell wall, contributing to the selective nature of lysin (López et al., 1997; Loessner et al., 2002).

The lytic spectrum of a lysin ranges from species-specific to targeting multiple genera. There are lysins that specifically target a single bacterial species [e.g., LysEF-P10 for *Enterococcus faecalis* (Cheng et al., 2017), and PlyPSA for *Listeria monocytogenes* (Zimmer et al., 2003; Schmelcher et al., 2011)] while some target various bacterial species of the same genus [e.g., LysK (O’Flaherty et al., 2005)]. Interestingly, several lysins have been identified to target multiple genera (Deutsch et al., 2004; Yoong et al., 2004; Gilmer et al., 2013). For example, PlyV12 targets Enterococci, Staphylococci, and Streptococci (Yoong et al., 2004), and PlySs2 lyses staphylococci, streptococci and Listeria (Gilmer et al., 2013).

The modular architecture of Gram-positive lysin enables the construction of chimeric lysins with enhanced lytic spectrum and lytic activity (Loessner et al., 2002; Schmelcher et al., 2011; Yang et al., 2014a; Dong et al., 2015; Broendum et al., 2018). For example, a chimeric lysin Ply187N-V12C exhibited a broader lytic spectrum from only targeting staphylococci to also kill Enterococci and streptococci after fusing the CHAP domain of Ply187 with the CBD of PlyV12 (Dong et al., 2015). While the CBD largely determines the lytic spectrum by recognizing specific cell wall components on the bacteria, the CD dictates the type of peptidoglycan bond to be cleaved (Nelson et al., 2012; Díez-Martínez et al., 2013; Oliveira et al., 2013; Broendum et al., 2018). The dependence of CBD for target recognition can be circumvented by tweaking the net charge of a lysin. A positively charged CD-only lysin was shown to maintain its lytic activity while possessing a broader lytic spectrum (Low et al., 2011). Bactericidal activity can be improved by fusing the CBD of a streptococcal lysin with the CD of a different lysin, or by modifying the linker between CD and CBD (Yang et al., 2019, 2020).

While there are many reports that successfully extended the lytic spectrum of a lysin (Schmelcher et al., 2011; Díez-Martínez et al., 2013; Dong et al., 2015; Broendum et al., 2018), few have managed to introduce species specificity in a chimeric lysin. In this study, we created two chimeric lysins by interchanging the CBDs of two parent lysins: PlyV12, a broad-spectrum lysin that target multiple genera (Yoong et al., 2004), and LysEF-P10, a narrow-spectrum lysin that only targets *E. faecalis* (Cheng et al., 2017). The lytic spectra of these lysins were characterized by subjecting them to a panel of Enterococcal and staphylococcal strains. The morphological changes, membrane integrity and cell viability in lysin-treated *Enterococcus faecium* cells were also evaluated. Comparisons between the chimeric lysins and the parent lysins enable a systematic elucidation of the respective roles of CD and CBD in determining the final lytic spectrum, thereby guiding the design and development of more selective chimeric lysins.

## Results

### Construct Design and Expression of Recombinant Lysins

**FIGURE 1:**
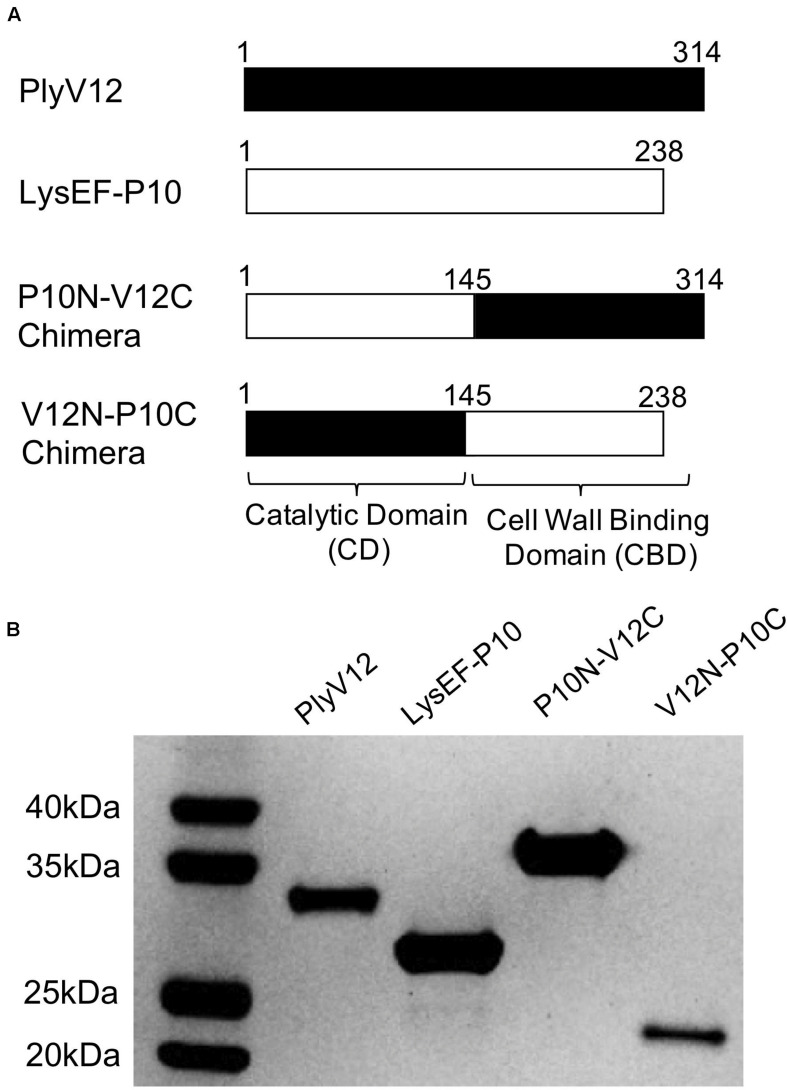
Description of the parent and chimeric recombinant lysins. **(A)** Schematic diagram illustrating the domain swapping between the parent lysins to create the two chimeras. The N-terminal domain is the catalytic domain whereas the C-terminal domain is the cell wall binding domain. **(B)** The SDS-PAGE analysis showed the high purity of the produced lysins. PlyV12 (35.0 kDa), P10N-V12C (35.4 kDa), V12N-P10C (27.2 kDa) contain a C-terminal 6xHis tag, while LysEF-P10 (29.1 kDa) contains an N-terminal 6xHis-TEV for affinity chromatography purification.

Two native lysins with different lytic spectra were selected as the parent lysins to generate chimeric lysins. LysEF-P10 is a narrow-spectrum lysin that only target *E. faecalis* (Cheng et al., 2017), whereas PlyV12 is a broad-spectrum lysin that targets Enterococci, staphylococci, and streptococci (Yoong et al., 2004). Two chimeric lysins, namely P10N-V12C and V12N-P10C, were created by swapping the CBD at the C-terminal domain of the two parent lysins (Figure 1A). The amino acid sequences of the chimeric lysins are listed in Supplementary Table S1. All 4 lysins were successfully expressed and purified (Figure 1B). The protein bands corresponding to PlyV12 and V12N-P10C are slightly lower than their molecular weights, which is not unusual as such observations have been reported with other lysins (Wittmann et al., 2010; Guo et al., 2017). It is worth noting that the high yield of PlyV12 lysin at 10 mg/L, is contrary to what previously reported (Yoong et al., 2004). The increased protein yield can be attributed to the recombinant gene with its codon usage optimized for *Escherichia coli* expression.

### Lysis Characterization at Various pH and Salt Concentrations

**FIGURE 2:**
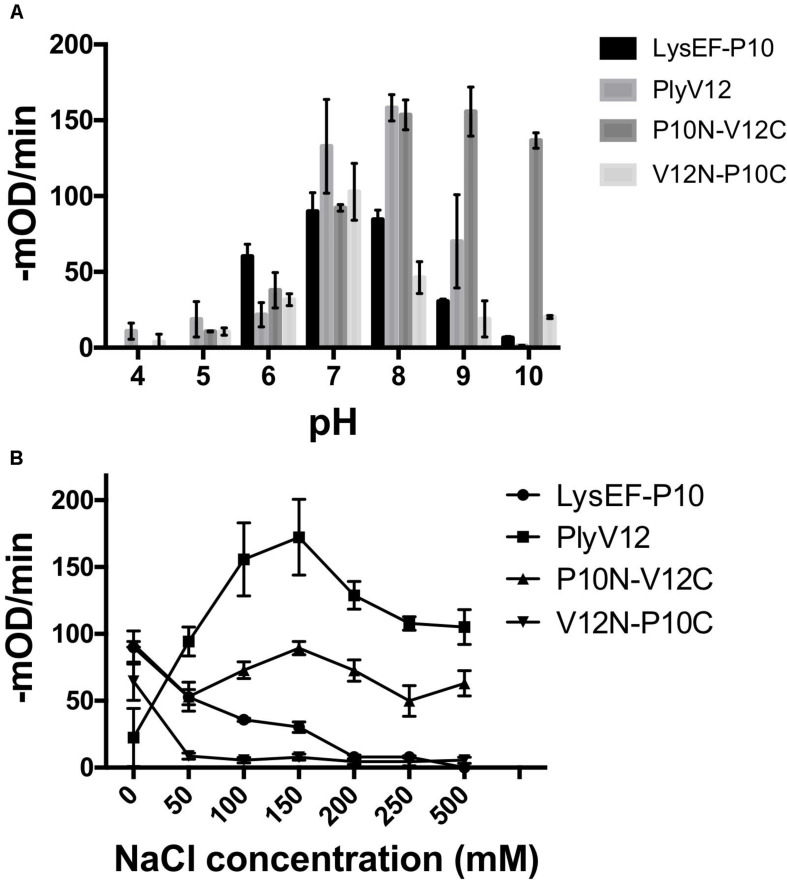
Characterization of lysins. The lytic activities of the four lysins at various pH buffers **(A)** under different salt concentrations **(B)**. These experiments were done in biological triplicates; error bars represent standard deviation.

The lytic activities of lysins can vary significantly with pH and salinity (Yoong et al., 2004; Dong et al., 2015; Swift et al., 2016; Cheng et al., 2017). Therefore, we tested the lysins against *E. faecalis* OG1RF strain in buffers of pH 4 to pH 10 and a series of salt concentrations ranging from 0 to 500 mM NaCl. We observed that P10N-V12C chimeric lysin maintains its lytic activity over a broader pH range, especially at pH 10, than the parent lysins, whereas LysEF-P10 and V12N-P10C share pH 7 as the optimal pH for lytic activity (Figure 2A). When tested in buffer with increasing salt concentrations, the lytic activities of LysEF-P10 and V12N-P10C reduced significantly with negligible activity in buffers with above 200 mM NaCl (Figure 2B). However, PlyV12 showed an opposite trend with low activity at 0 mM NaCl and maximum activity at 150 mM NaCl. Although the lytic activity of PlyV12 decreases slightly from 200 mM NaCl onward, PlyV12 is still active at >100 –mOD/min. Interestingly, P10N-V12C chimera exhibited a superior salt tolerance than both of its parent lysins as it retains its lytic activity from 0 to 500 mM NaCl.

### Dose Response and Lytic Kinetics of Various Lysins

**FIGURE 3:**
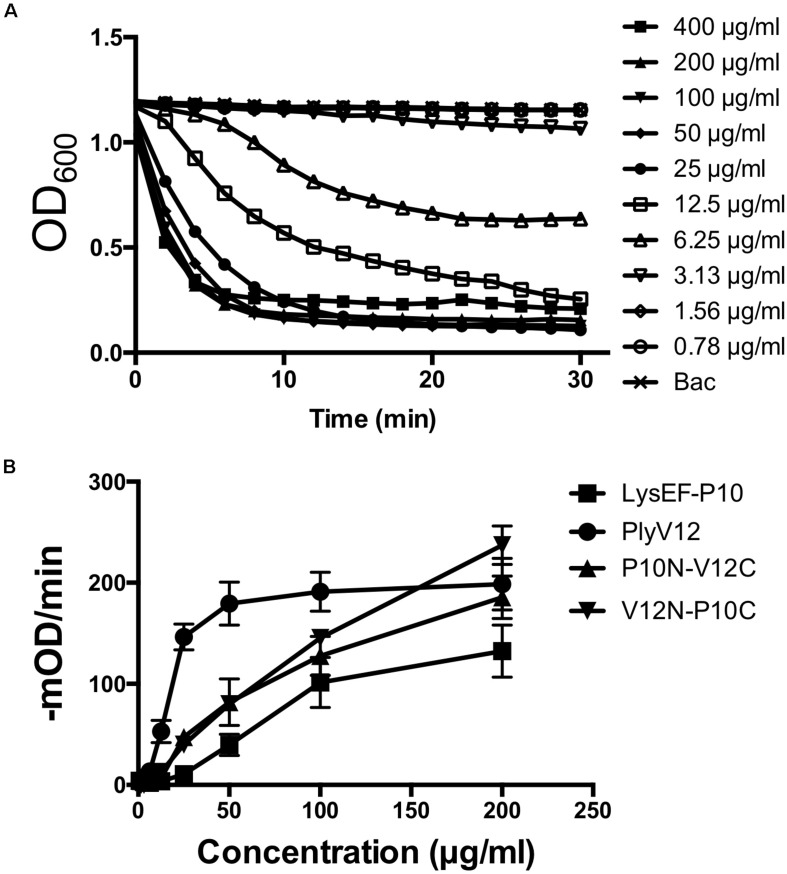
Lytic activity characterization. **(A)** Lytic kinetics of PlyV12 against *E. faecalis* OR1GF strain at various doses as measured by the reduction in OD_600_. **(B)** Lytic activity at various doses as measured by the rate of reduction in OD_600_ in the first 3 min. These experiments were performed in biological triplicates; error bars represent standard deviation.

To determine the optimal doses of lysins for subsequent experiments, we measured the lytic activities of all 4 lysins against *E. faecalis* OG1RF strain by performing turbidity reduction assays. While PlyV12 requires at least 25 μg/ml to achieve > 90% reduction in OD_600_ in 30 min (Figure 3A), the other 3 lysins require 100 μg/ml (Supplementary Figure S1). At these lysin concentrations, the lytic activities are optimal as indicated by their high lytic rates of over 100-mOD/min (Figure 3B). Therefore, 25 μg/ml for PlyV12 and 100 μg/ml for LysEF-P10, P10N-V12C, and V12N-P10C were selected to further characterize the lysins.

### Lytic Spectra and Bactericidal Activities of Lysins

**FIGURE 4:**
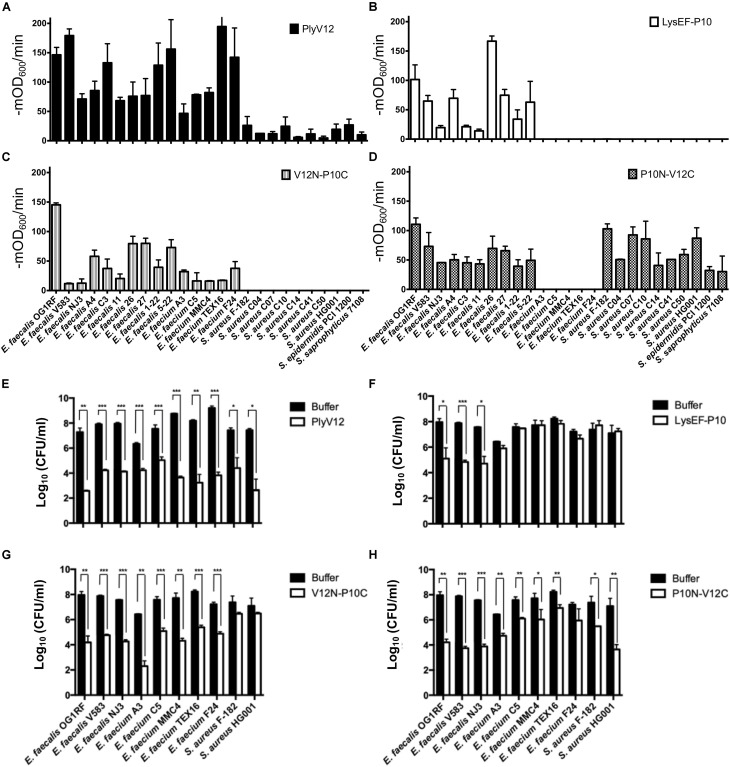
Lytic spectra and bactericidal activities of the parent and chimeric lysins. **(A–D)** The lytic activities of these four lysins against 15 enterococcal and 10 staphylococcal strains were measured as the reduction in OD_600_ per minute in the first 3 min. **(E–H)** 3 *E. faecalis*, 5 *E. faecium* and 2 *S. aureus* strains were treated with the four lysins under their respective optimal conditions. The number of residual CFU in each treatment was determined by plating 10-fold serial dilution and compared to that of buffer-treated controls by a two-tailed Student’s *t*-test with Welch’s correction. *P < 0.05; **P < 0.01; ***P < 0.001. Experiments were done in biological triplicate; error bars represent standard deviation.

To check whether the lytic spectrum of a lysin is dictated entirely by its CBD, we swapped the CBD of a species-specific lysin (P10C) with the CBD of a broad-spectrum lysin (V12C), and vice versa. The resulting chimeric lysins, P10N-V12C and V12N-P10C, along with the parent lysins, LysEF-P10 and PlyV12, were tested on 15 enterococcal and 10 staphylococcal strains, including 5 vancomycin-resistant enterococci (VRE) and 7 methicillin-resistance *Staphylococcus aureus* (MRSA) (Table 1). PlyV12 targets all enterococcal and staphylococcal strains (Figure 4A). PlyV12 shows a slight preference toward enterococci than staphylococci as illustrated by the higher lytic activities on enterococci, which is consistent with Yoong et al. (2004). LysEF-P10 only targets the *E. faecalis* strains (Figure 4B), with a lytic profile that is consistent with the previous study (Cheng et al., 2017).

**TABLE 1:** Bacterial strains used in this study and their susceptibility to the 4 lysins tested.

Bacterial strains	Characteristics	Reference or source	Presence of lytic activity observed on^c^			
			PlyV12	LysEF-P10	P10N-V12C	V12N-P10C
**Enterococci**						
*E. faecalis* OG1RF	Rifampicin resistant	ATCC 47077, (Dunny et al., 1978)	+	+	+	+
*E. faecalis* V583	Vancomycin resistant	ATCC 700802, (Paulsen et al., 2003)	+	+	+	+
*E. faecalis* NJ3	Vancomycin resistant	ATCC 51299	+	+	+	+
*E. faecalis* A4	Isolate from healthy child gut	WUSTL^a^, (Denno et al., 2012)	+	+	+	+
*E. faecalis* C3	Isolate from healthy child gut	WUSTL^a^, (Denno et al., 2012)	+	+	+	+
*E. faecalis* 11	Non-VRE clinical wound isolate	TTSH^b^	+	+	+	+
*E. faecalis* 26	Non-VRE clinical wound isolate	TTSH^b^	+	+	+	+
*E. faecalis* 27	Non-VRE clinical wound isolate	TTSH^b^	+	+	+	+
*E. faecalis* 1–22	VRE clinical blood isolate	WUSTL^a^, (Denno et al., 2012)	+	+	+	+
*E. faecalis* 5–22	VRE clinical blood isolate	WUSTL^a^, (Denno et al., 2012)	+	+	+	+
*E. faecium* A3	Isolate from healthy child gut	WUSTL^a^, (Denno et al., 2012)	+	–	–	+
*E. faecium* C5	Isolate from healthy child gut	WUSTL^a^, (Denno et al., 2012)	+	–	–	+
*E. faecium* MMC4	Rifampicin and vancomycin resistant	ATCC 51559	+	–	–	+
*E. faecium* TEX16	Clinical blood isolate	ATCC BAA-472	+	–	–	+
*E. faecium* F24		ATCC 25307	+	–	–	+
**Staphylococci**						
*S. aureus* F-182	Methicillin resistant	ATCC 43300	+	–	+	–
*S. aureus* C04	MRSA clinical wound isolate	TTSH^b^	+	–	+	–
*S. aureus* C07	MRSA clinical wound isolate	TTSH^b^	+	–	+	–
*S. aureus* C10	MRSA clinical wound isolate	TTSH^b^	+	–	+	–
*S. aureus* C14	MRSA clinical wound isolate	TTSH^b^	+	–	+	–
*S. aureus* C41	MRSA clinical wound isolate	TTSH^b^	+	–	+	–
*S. aureus* C50	MRSA clinical wound isolate	TTSH^b^	+	–	+	–
*S. aureus* HG001	Methicillin susceptible	(Caldelari et al., 2017)	+	–	+	–
*S. epidermidis* PCI 1200	Non-pathogenic commensal	ATCC 12228	+	–	+	–
*S. saprophyticus* 7108	Wild-type strain	(Gatermann et al., 1988)	+	–	+	–

*^a^WUSTL: Washington University in St. Louis, MO, United States. ^b^TTSH: Tan Tock Seng Hospital, Singapore. ^c^The presence (+) and absence (−) of lytic activity as measured by the turbidity reduction assay (Figure 4).*

The bactericidal activities of the lysins were determined for 3 *E. faecalis*, 5 *E. faecium* and 2 *S. aureus* strains. PlyV12 effectively kills all bacteria tested whereas LysEF-P10 only exhibited significant killing on *E. faecalis* strains (Figures 4E,F). The chimeric lysin V12N-P10C kills all enterococcal strains tested while P10N-V12C exhibit over 3-log CFU reduction on *E. faecalis* and *S. aureus* and over 1-log CFU reduction on *E. faecium* (Figures 4G,H).

Swapping the broad-spectrum CBD from PlyV12 with a CBD from the narrow-spectrum LysEF-P10 lysin, the V12N-P10C chimera lyses both *E. faecalis* and *E. faecium*, but it does not target staphylococci (Figure 4D), suggesting the CBD is crucial in distinguishing bacteria of different genera. For the chimeric lysin with the CBD of PlyV12, P10N-V12C can target all staphylococcal strains and all *E. faecalis* strains (Figure 4C). However, it does not lyse *E. faecium*. This was surprising because a previously reported chimeric lysin P187N-V12C, which also carries the CBD of PlyV12, was shown to lyse all of the tested streptococcal, staphylococcal, and enterococcal strains, including *E. faecium* (Dong et al., 2015). Considering that the only difference between P187N-V12C and our P10N-V12C chimeric lysin is the catalytic domain, we deduced that the catalytic domain in P10N-V12C is responsible for distinguishing *E. faecium* from *E. faecalis*.

### The Effect of P10N-V12C Lysin on *E. faecium*

**FIGURE 5:**
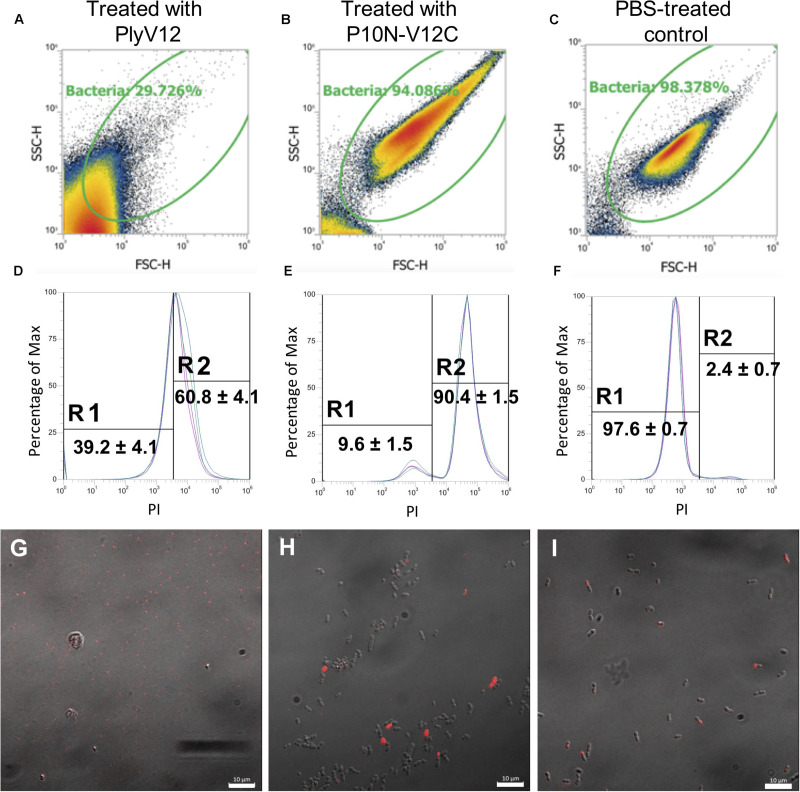
The effect of lysin treatment on *E. faecium*. FSC-SSC dot plots of *E. faecium* strain A3 cells upon treated with PlyV12 **(A)**, P10N-V12C **(B)**, and PBS treatment **(C)**. **(D–F)** The corresponding histogram displaying percentage of gated cells with PI uptake. PI-negative and PI-positive cells are labeled with R1 and R2, respectively. Confocal microscopy images of the *E. faecium* cells with treatment of PlyV12 **(G)**, P10N-V12C **(H)** and PBS as control **(I)**. Cells with PI uptake were detected as red fluorescence signals. White bars indicate 10 μm.

To investigate why P10N-V12C does not lyse *E. faecium*, despite carrying a broad-spectrum CBD, lysin-treated *E. faecium* cells were further analyzed using flow cytometry and confocal microscopy. We first observed that PlyV12 effectively lysed most *E. faecium* strain A3 cells as reflected by the low number of intact cells (Figure 5A) and the 2-log CFU reduction (Figure 4E). The remaining PlyV12-treated cells are less viable as reflected by the high PI uptake compared to the PBS-treated control (Figures 5D,F). The forward-scattered (FSC) and side-scattered (SSC) values of the PlyV12-treated cells (Figure 5A) were drastically reduced compared to the PBS-treated control (Figure 5C). This reduction can be attributed to the cell debris and aggregates formed upon cell lysis. Additionally, significantly fewer bacterial cells were visible by confocal microscopy. Instead of individual intact cells, aggregates of presumably dead cells were observed upon PlyV12 treatment (Figure 5G). By contrast, the P10N-V12C-treated *E. faecium* cells (Figure 5B and Supplementary Figure S2) were more elongated and slightly larger in overall size based on their FSC/SSC profiles compared to the buffer-treated controls (Figure 5C). These bacterial cells are still intact with a similar overall morphology as the PBS-treated control under the microscope (Figures 5H,I). However, a majority of these P10N-V12C-treated bacterial cells are compromised and less viable as shown by the significant uptake of PI dye as measured by flow cytometry (Figure 5E) and over 1-log CFU reduction (Figure 4H). The loss in cell viability is likely due to the membrane is compromised by the lysins bound to the cell wall as the CBD of PlyV12 is known to bind to *E. faecium* (Dong et al., 2015). Unlike PlyV12 which kills *E. faecium* by cell lysis, the chimeric lysin P10N-V12C kills the *E. faecium* cells by binding to the cell wall and compromising the bacterial membrane with its CBD. The lack of lytic activity of P10N-V12C on *E. faecium* (Figures 4D, 5H) is due to its species-specific CD does not hydrolyze the peptidoglycan of *E. faecium*.

## Discussion

With the emergence of multidrug-resistant bacteria, including VRE and MRSA, there is an urgent need to develop new antibacterial therapeutics. Lysins are phage-derived peptidoglycan hydrolases that kill bacteria selectively and quickly. They can be customized and engineered to create novel lysins by swapping their modular domains. As the lytic spectrum is mostly dictated by the CBD, a lysin can be engineered to only target a selected few bacterial species or target multiple bacterial genera by modifying its CBD.

In this study, we constructed two chimeric lysins by swapping the CBD of two parents lysins with different properties. We found that domain-swapping affects the functional pH and salinity tolerance of the two chimeric lysins. P10N-V12C outperformed its parent lysins by maintaining high lytic activities across a broader range of pH and salt concentrations. However, the other chimeric lysin, V12N-P10C, has limited pH range with diminished activity in the presence of salt. These observations show the importance of CBD in determining the optimal pH and salt concentration where a lysin is most active at.

The two parent lysins selected in this study have different lytic spectra: LysEF-P10 only targeting *E. faecalis* and PlyV12 targeting multiple genera. Upon swapping the CBD of LysEF-P10, the lytic spectrum of V12N-P10C was narrowed as it only targets enterococci. Conversely, P10N-V12C exhibits a broadened lytic spectrum with the CBD of PlyV12. Interestingly, although P10N-V12C now targets staphylococcal strains, it retains the species-specificity feature of LysEF-P10 on *E. faecalis* and does not lyse *E. faecium*. Interestingly, despite the lack of lytic activity on *E. faecium*, P10N-V12C still exhibited killing with over 1-log CFU reduction. It is worth noting that without a lytic activity on *E. faecium*, the bactericidal activity of P10N-V12C on *E. faecium* is less significant as compared to the 3–4 log CFU reduction observed on *E. faecalis.* These data suggest that the CD also contributes, albeit to a lesser extent compared to the CBD, to the final lytic spectrum and bactericidal activity of a chimeric lysin.

Flow cytometry and confocal microscopy revealed that the *E. faecium* cells that have been treated with P10N-V12C have a compromised membrane, but the cells remain intact. It was previously shown that GFP-fused CBD of PlyV12 binds to staphylococcal and enterococcal cells, including *E. faecium* (Dong et al., 2015), therefore the CBD of P10N-V12C is expected to bind to the cell wall of *E. faecium*. This could explain the slightly larger and elongated *E. faecium* cells upon exposure of P10N-V12C. Such binding event likely disrupts the bacterial membrane that leads to the PI uptake of the cells, thereby compromising the cell viability. However, the treated cells were not lysed as the CD of P10N-V12C does not hydrolyze the peptidoglycan of *E. faecium*.

A possible explanation for how the CD of P10N-V12C can distinguish the peptidoglycan of *E. faecium* from *E. faecalis* and *S. aureus* is that this CD contains a cysteine, histidine-dependent amidohydrolases/peptidases (CHAP) domain which cleaves the peptide bonds of the pentaglycine bridge-links connecting the stem peptides. Some CHAP domains can also target the amide bonds connecting the glycan and peptide moieties of the peptidoglycan (Broendum et al., 2018; Vermassen et al., 2019). On the contrary, the CD of V12N-P10C belongs to the class of N-acetylmuramoyl-L-alanine amidase that only targets the amide bond connecting the sugar and peptide components of the peptidoglycan (Love et al., 2020). Many selective lysins contain a CHAP domain. For example, both LysEF-P10 and VD13 lysins, which specifically target *E. faecalis*, contain a CHAP domain (Swift et al., 2016; Cheng et al., 2017). A crystallographic study on LysK, a *Staphylococcus*-specific lysin (O’Flaherty et al., 2005), identified a catalytic triad at the CHAP domain that targets a cross-bridge peptide bond unique to Staphylococci (Becker et al., 2009; Szweda et al., 2012; Sanz-Gaitero et al., 2014). Considering most of the diversity in the peptidoglycan composition occurs with the nature of the crosslinking of the stem peptides (Schleifer and Kandler, 1972; Vollmer et al., 2008; Vermassen et al., 2019), we suggest that lysins with CHAP domain are likely to be more selective and target-specific than lysins containing other classes of peptidoglycan hydrolases.

## Conclusion

In conclusion, we introduced species specificity to an otherwise, broad-spectrum lysin by swapping its catalytic domain with that of a species-specific lysin. The resulting chimeric lysin P10N-V12C selectively lyses *E. faecalis* over *E. faecium* and kills *E. faecalis* more effectively than *E. faecium* while it remains active against all staphylococcal strains tested. It possesses a high lytic activity across a broader range of pH and salt concentrations than any of the parent lysins. While CBD swapping helps to extend the lytic spectrum such that multiple bacterial genera can be targeted, choosing the correct CD to fuse with the CBD is important to achieve species specificity in a particular genus. With more understanding of the roles of both CD and CBD, novel lysins can be tailor-made to develop a highly targeted antimicrobial therapy.

## Materials and Methods

### Cloning, Expression, and Purification of Phage Lysins

Lysin genes PlyV12 (GenBank accession No. AAT01859.1) and LysEF-P10 (GenBank accession No. AQT27695.1) were synthesized and cloned into pNIC-CH and pNIC28-Bsa4 vectors by Bio Basic Inc. The nucleotide sequences were codon-optimized to improve the efficiency of soluble expression in *E. coli*. Chimeric lysin genes of P10N-V12C and V12N-P10C were produced using overlap extension PCR using the primers listed in Supplementary Table S1.

To produce the 2 chimeric lysins, the C-terminal cell wall binding domain (CBD) of PlyV12 and LysEF-P10 were amplified as inserts for P10N-V12C and V12N-P10C, respectively. The inserts were purified and extended using primers containing the sequences of the N-terminal CD and the C-terminal his-tag in the vector pNIC-CH. The extended PCR products were used as megaprimers to swap the CBD of PlyV12 and LysEF-P10, creating the two chimeric lysins. After confirmed by sequencing, the plasmids were transformed into competent cells of BL21(DE3)-T1R *E. coli* Rosetta strain for efficient protein expression.

To produce the recombinant lysins, the transformed *E. coli* was incubated at 37°C in LB broth containing 34 μg/ml chloramphenicol and 50 μg/ml kanamycin until the culture reaches mid-log phase (OD_600_ 0.5–0.6). After cooling the culture down, protein overexpression was induced by adding 0.2 mM isopropyl β-D-thiogalactoside and incubated at 16°C for 16–18 h. Cells were harvested by centrifugation at 4,000 × *g* for 25 min at 4°C and the cell pellet was stored at −80°C until ready to purify.

To purify the proteins, the cell pellets were resuspended with lysis buffer before being disrupted by sonication on ice. PlyV12, chimeric lysins P10N-V12C and V12N-P10C used lysis buffer at pH 7.5 (50 mM HEPES pH 7.5, 500 mM NaCl, 5% glycerol, 0.5 mM DTT), whereas LysEF-P10 used lysis buffer at pH 8.5 (50 mM Tris pH 8.5, 500 mM NaCl, 5% glycerol, 0.5 mM DTT). Cell debris was removed by centrifugation for 1 h at 40,000 × *g* at 4°C to obtain the soluble protein. The supernatant was sterile filtered and mixed with 2 mL of nickel-nitrilotriacetic acid (Ni-NTA) resin and was incubated for 2 h at 4°C in a tube rotator. The desired proteins were collected by washing and eluting with lysis buffers containing 0, 10, 20, 250, and 500 mM imidazole. The purity of the protein was evaluated using SDS-PAGE gel electrophoresis and the gel stained with Coomassie blue. Finally, the collected fractions were subjected to buffer exchange via diafiltration to reduce the imidazole concentration to <10 mM and concentrated to >5 mg/ml before storing at −80°C.

### Characterization of Lytic Activities and Spectra of Recombinant Lysins

Turbidity reduction assay was performed in 96-well microtiter plate to measure the lytic activity of the lysins produced (Nelson et al., 2001; Yang et al., 2014b). Enterococcal and staphylococcal strains were grown to mid-logarithmic phase in brain heart infusion (BHI) and Mueller Hilton (MH) broths, respectively. The bacteria were then pelleted and resuspended in assay buffers to an OD_600_ of 1.0−1.2. Lysin was added to the bacteria with a volume ratio of 1:4 to a final volume of 100 μl. To profile the optimal pH and salt concentrations of the lysins, *E. faecalis* OG1RF strain was selected as this strain is well-characterized with its genome sequenced. Additionally, all 4 lysins are expected to exhibit lytic activities against *E. faecalis*. The pH profiling experiments were performed after the bacterial cells were resuspended in 20 mM acetate buffer at pH 5 and 6, 20 mM of phosphate buffer at pH 6, 7, 8, and 20 mM Tris buffer at pH 9, 10. The activities of the 4 lysins were also characterized under NaCl concentrations ranging from 0 to 500 mM. For dose response assays, 10 lysin concentrations of 0.78 to 400 μg/ml were tested. Upon identifying the optimal dose, 25 μg/ml of PlyV12 and 100 μg/ml of LysEF-P10, P10N-V12C, and V12N-P10C were used in the turbidity reduction assays. The lytic activity is measured by the reduction in OD_600_ per minute for the first 3 min (Yang et al., 2017). All assays were done with three biological replicates and all buffers were autoclaved before use. The bacterial OD_600_ was measured in a microplate reader (BioTek Synergy 4 Plate Reader) for an hour with occasional shaking at 40-s intervals. To enumerate the residual colony-forming units (CFU) of the lysin-treated bacterial cells, a similar turbidity reduction assay was performed with a starting OD_600_ of 0.05 (∼10^8^ CFU/ml). After the 1-h incubation, these lysin-treated bacteria were serially diluted and plated on BHI and MH agar accordingly. As a negative control, the respective bacterial strains were treated with equivalent quantity of buffer solutions.

### Flow Cytometry Analysis of Lysin-Treated Bacterial Cells

*Enterococcus faecium* strain A3 was grown to mid-logarithmic phase (OD_600_ = 0.5) in BHI. The bacteria were then pelleted and resuspended in 10% BHI diluted with 1× PBS, to an OD_600_ of 0.08 (∼10^9^ CFU/ml). Lysins PlyV12 and P10N-V12C were added to the resuspended bacterial cell on a 1:1 ratio such that the starting OD is 0.04 and the final concentrations of lysins are 25 μg/ml (PlyV12) and 100 μg/ml (P10N-V12C), respectively. After 1-h incubation, 2 μg/ml of Propidium Iodide (PI) was added and incubated in the dark for 15 min at room temperature.

Flow cytometry analyses were performed using Attune NxT 4 lasers and 14 colors system, flat top laser from Thermo Fisher Scientific. Sample delivery utilizing positive-displacement syringe pump for direct volumetric analysis provides concentration measurement without using counting beads. The 96-well U-bottom sample plate was loaded and run with Attune NxT autosampler without prior washing. Total sample volume was 105 μl and acquisition volume was 40 μl. Data acquisition were set at flow rate of 25 μl/min with 2 mixing cycles and 3 rinsing cycles. Total events acquired were 100,000. 25 μl/min speed was the acquisition guideline provided by Thermo Fisher Scientific, with maximum sample concentration of not more than 5.4 × 10^7^/ml. PI was excited by 561 nm yellow Laser and fluorescent emission were detected at 620/15 band pass emission filter at YL2 PMT detector. The threshold was set at FSC (0.8 × 1000) and SSC (1.0 × 1000). Data were collected and analyzed using Attune NxT software. FSC and SSC voltage were set based on unstained healthy bacteria samples. Heat killed bacteria samples were used as a positive control for PI single staining (Supplementary Figure S3).

### Bacterial Visualization Using Confocal Microscopy

The *E. faecium* cells were treated with lysins using the same protocol as the sample preparation for the flow cytometry analysis. The lysin-treated and untreated *E. faecium* cells were imaged using confocal laser scanning microscopy (CLSM). Propidium Iodine (PI) was added to all sample at a final concentration of 2 μg/ml for 15 min in the dark, similar to the flow cytometry experiments. Bacterial cells that are dead or have compromised membrane will uptake PI and appear red under CLSM. Confocal images were acquired on a Zeiss LSM700 CLSM system under bright field light and fluorescence using 40× and 63× oil-immersion objectives. PI was excited at 555 nm and fluorescence emission were detected at 573 nm. Images were then processed using ZEN software (version 14.0).

### Statistical Analyses

Data were analyzed by using two-tailed Student’s *t*-test with Welch’s correction, and a value of *P* < 0.05 was considered statistically significant, (*P < 0.05; **P < 0.01; ***P < 0.001).

## Data Availability Statement

The original contributions presented in the study are included in the article/Supplementary Material, further inquiries can be directed to the corresponding author.

## Author Contributions

BG conceived, designed, and supervised the study. HB, LT, JK, LD, and HL performed the experiments. HB, LT, JK, LD, HL, and BG analyzed the data, prepared the tables and figures, and drafted the manuscript. KK contributed bacterial strains and provided advice and suggestions. KK and BG revised the manuscript. All authors have read and approved the submission of the manuscript.

## Conflict of Interest

The authors declare that the research was conducted in the absence of any commercial or financial relationships that could be construed as a potential conflict of interest.
