# Supplementary material for: Engineered Lysins With Customized Lytic Activities Against Enterococci and Staphylococci
Source: Front Microbiol. 2020 Nov 25;11:574739. doi: 10.3389/fmicb.2020.574739 (PMC7724435; doi:10.3389/fmicb.2020.574739)
Supplement: Supplementary file 1 [file Data_Sheet_1.PDF]

## Supplemental materials

**Table S1**: Primer designs to create the chimeric lysins and the final amino acid sequences of the two chimeric lysins

| Target gene | Primer | Sequence (5' to 3') |
|---|---|---|
| *PlyV12 insert* | plyV12 fwd | CTGAACGGTGGTTCTACCCC |
| | plyV12 rev | TTTGAAGGTACCCCACGCTTC |
| *PlyV12 chimeric insert* | P10N-V12C fwd | CGTCCGCCGTACGAAAAAGATACCCCGCTGAACGG TGGTTCTACCCC |
| | P10N-V12C rev | GGATCCTCAATGGTGGTGATGATGGTGCGCTTTGA AGGTACCCCACGCTTC |
| *LysEF-P10 insert* | LysEF-P10 fwd | GCGCCGAAACCGCCGG |
| | LysEF-P10 rev | CGCAACTTTGAACTGCGGGTGAGACAG |
| *LysEF-P10 chimeric insert* | V12N-P10C fwd | GTATAGCATGGGTTGGTACGTTTATCGTCTGGCGC CGAAACCGCCG |
| | V12N-P10C rev | GGATCCTCAATGGTGGTGATGATGGTGCGCAACTT TGAACTGCGGGTGAGACA |
| Chimeric lysin P10N-V12C | | MVKVNDVVSYLNSRVGTGIDMDGAYGFQCADLAQAIT YNFFGWWFYGNAIALASQPIPNGFERIRVTDATQIKA GDIVVWSEHEYAQYGHVAIAAKDGYSDQTFENYAQNW LNASLTVGSPIALVRTNMYGVGYVIRPPYEKDTPLNG GSTPPKPNTKKVKVLKHATNWSPSSKGAKMASFVKGG TFEVKQQRPISYSYSNQEYLIVNKGTVLGWVLSQDIE GGYGSDRVGGSKPKLPAGFTKEEATFINGNAPITTRK NKPSLSSQTAIPLYPGQSVRYLGWKSAEGYIWIYATD GRYIPVRPVGKEAWGTFKAHHHHHH |
| Chimeric lysin V12N-P10C | | MSNINMETAIANMYALKARGITYSMNYSRTGADGTGD CSGTVYDSLRKAGASDAGWVLNTDSMHSWLEKNGFKL IAQNKEWSAKRGDVVIFGKKGASGGSAGHVVIFISST QIIHCTWKSATANGVYVDNEATTCPYSMGWYVYRLAP KPPAPKPAKPNLPTPKGDDTMLAIYYKHLKNGNVEQW LLINGKRVYLPTQTWVNEANALIKAYGGTKEVVQYNH DNFGLKLLELSHPQFKVAHHHHHH |

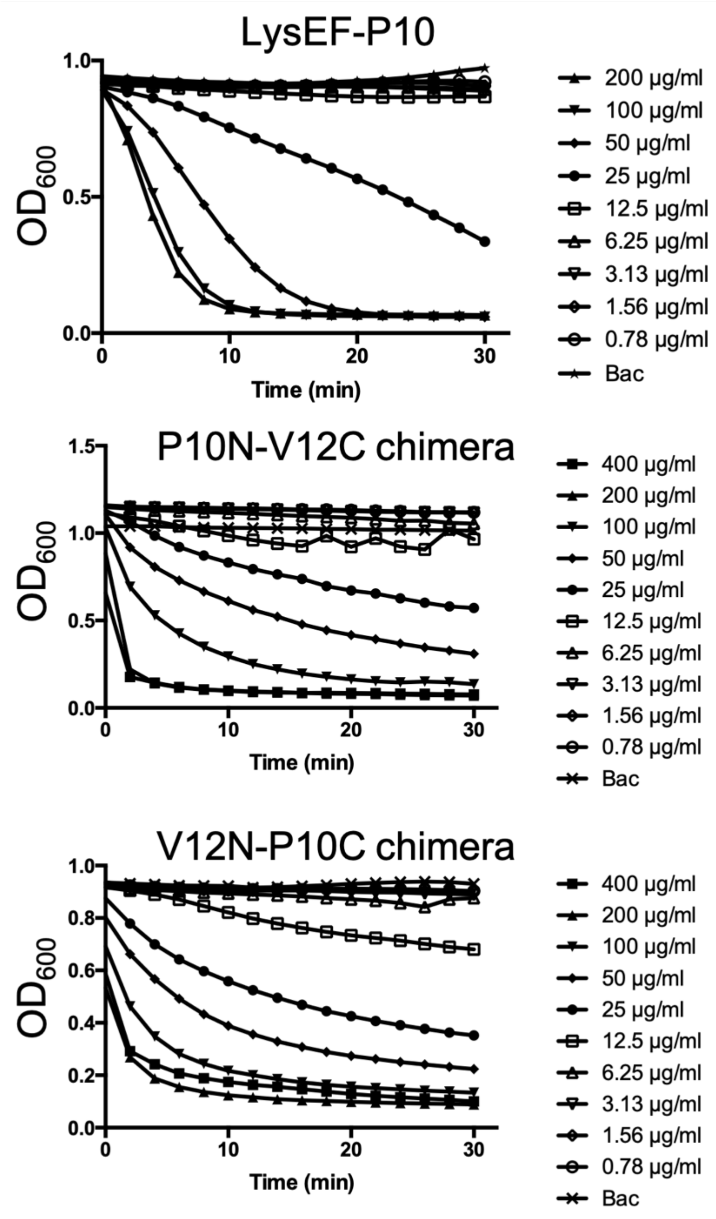


**Figure S1**. Dose response for other 3 lysins. Lysin concentrations that achieved >90% $OD_{600}$ reduction were used in this study.

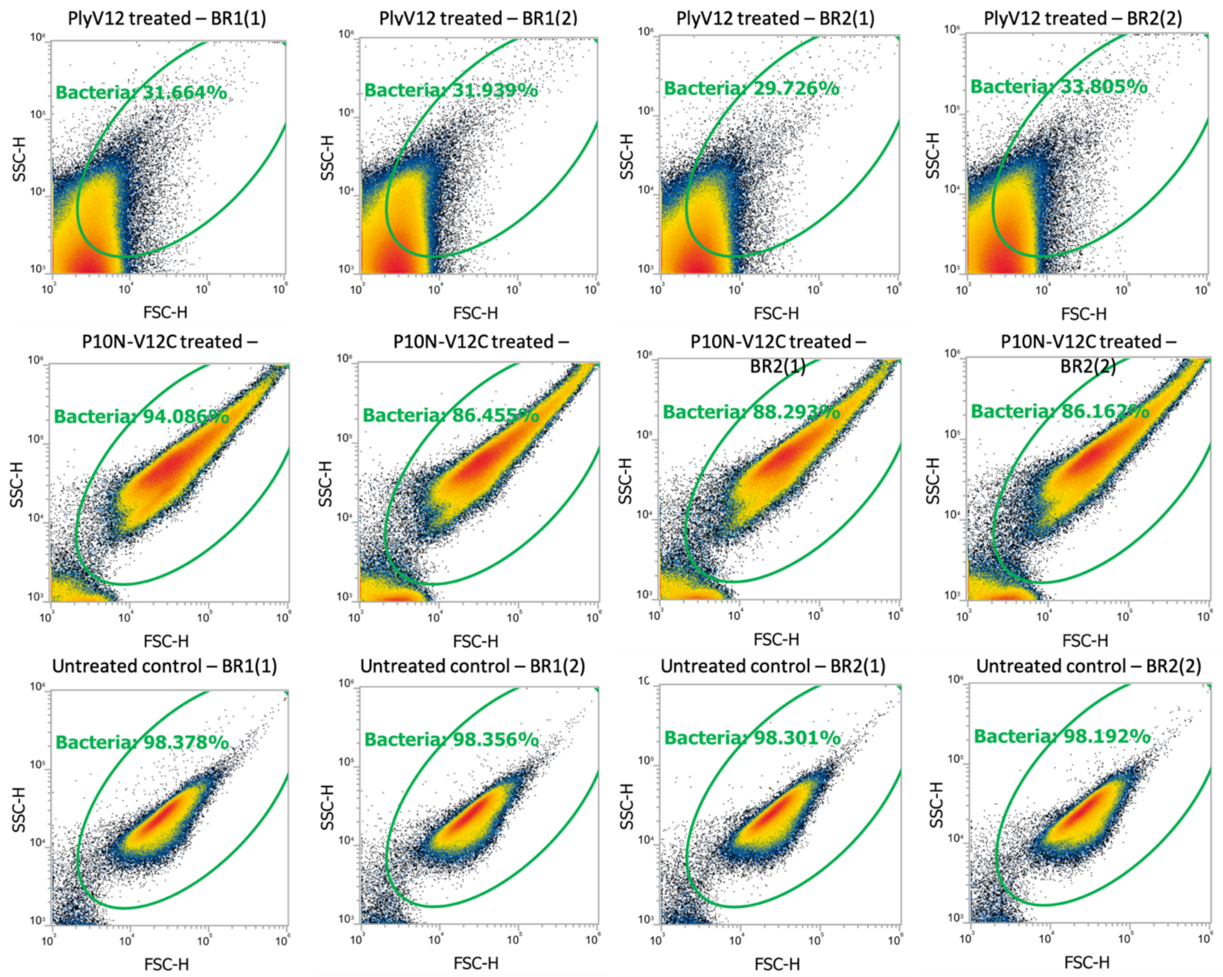


**Figure S2**. SSC-FSC plots of the 4 replicates that were performed. PlyV12 and P10N-V12C lysin treated E faecium cells are shown in the top and middle rows, respectively. The untreated control runs are shown in the bottom row. BR1(1) refers to biological replicate 1, technical duplicate 1.

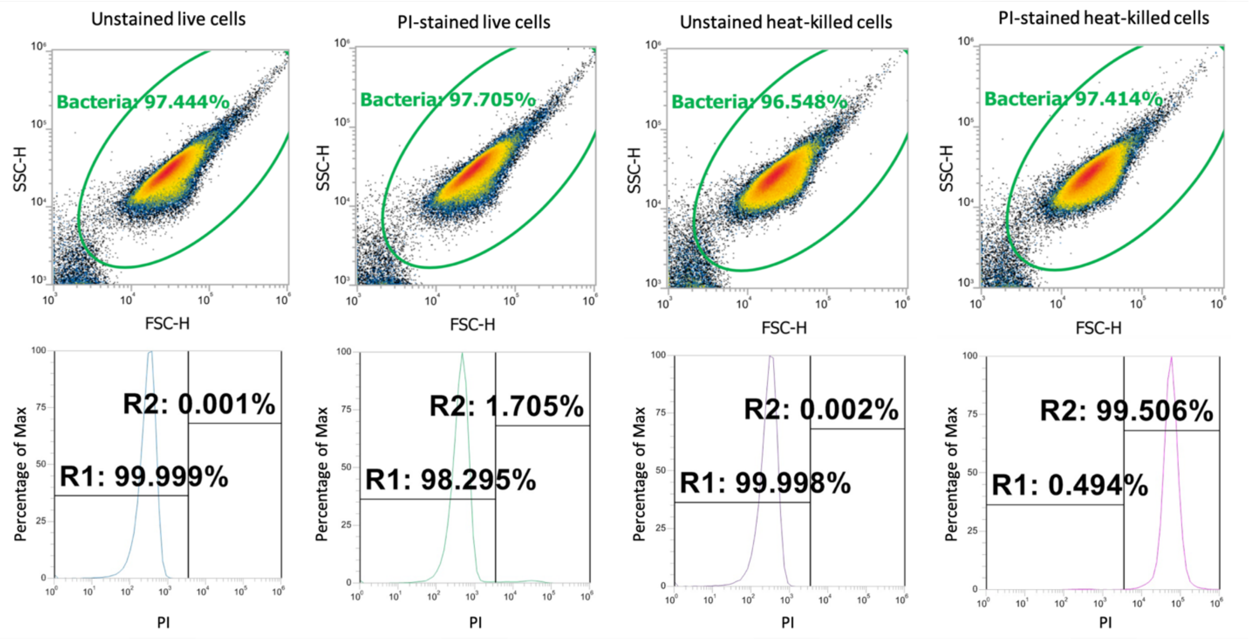


**Figure S3**. Controls for the flow cytometry experiments used to establish gating parameters. The FSC-SSC plots are at the top row, whereas the bottom row shows the percentage of gated cells with PI uptake. PI-negative and PI-positive cells are labeled with R1 and R2 respectively.
